# Microglia Polarization in Alzheimer’s Disease: Mechanisms and a Potential Therapeutic Target

**DOI:** 10.3389/fnagi.2021.772717

**Published:** 2021-11-08

**Authors:** Qinqin Wang, Hongmei Yao, Wenyan Liu, Bailiu Ya, Hongju Cheng, Zhenkai Xing, Yili Wu

**Affiliations:** ^1^Shandong Collaborative Innovation Center for Diagnosis, Treatment and Behavioral Interventions of Mental Disorders, Institute of Mental Health, Jining Medical University, Jining, China; ^2^Affiliated Hospital of Jining Medical University, Jining, China; ^3^Department of Physiology, College of Basic Medicine, Jining Medical University, Jining, China; ^4^The Affiliated Kangning Hospital, Wenzhou Medical University, Wenzhou, China; ^5^Key Laboratory of Alzheimer’s Disease of Zhejiang Province, Institute of Aging, School of Mental Health, Wenzhou Medical University, Wenzhou, China; ^6^Oujiang Laboratory, Wenzhou, China

**Keywords:** neuroinflammation, microglia activation, M1 microglia, M2 microglia, Alzheimer’s disease

## Abstract

Neuroinflammation regulated by microglia is one of the important factors involved in the pathogenesis of Alzheimer’s disease (AD). Activated microglia exhibited phenotypes termed as M1 and M2 phenotypes separately. M1 microglia contribute to the development of inflammation *via* upregulating pro-inflammatory cytokines, while M2 microglia exert anti-inflammation effects through enhancing the expression of anti-inflammation factors. Moreover, M1 and M2 microglia could be mutually transformed under various conditions. Both M1 and M2 microglia are implicated in AD. Amyloid-β (Aβ) and hyperphosphorylated tau are two major components of AD pathological hallmarks, neuritic plaques, and neurofibrillary tangles. Both Aβ and hyperphosphorylated tau were involved in microglial activation and subsequent inflammation, which further contribute to neuronal and synaptic loss in AD. In this review, we summarized the roles of M1 and M2 microglia in AD and underlying mechanisms, which will provide an insight into the role of microglia in the pathogenesis of AD and highlight the therapeutic potential of modulating microglia.

## Introduction

As a devastating age-related brain disorder, Alzheimer’s disease (AD) is characterized by progressive memory loss and cognitive deficits. Extracellular neuritic plaques, mainly consisting of amyloid-β (Aβ) and intracellular neurofibrillary tangles (NFTs) containing hyperphosphorylated tau, are the major pathological hallmarks of AD (Palomer et al., 2019; Wang Z. et al., 2019; Frolich, 2020). Although genetic factors, abnormal cholesterol metabolism, and protein homeostasis deficiency have been reported to contribute to AD pathogenesis (Karch and Goate, 2015; Liebsch et al., 2019; van der Kant et al., 2019; Wang and Davis, 2021), molecular mechanisms of AD remain elusive.

Emerging evidence suggested that neuroinflammation mediated by microglia was an important feature of AD (Griciuc et al., 2019; Paouri and Georgopoulos, 2019). Microglia were activated through a classic pathway or an alternative pathway, termed M1 or M2 microglia (Wang Y. et al., 2019). M1 and M2 microglia had different properties and functions (Wang Y. et al., 2019), which were differentially involved in the pathogenesis of AD (Colton et al., 2006; Paouri and Georgopoulos, 2019). Advanced understanding of the roles of M1 and M2 microglia in AD will provide new clues about the pathological mechanisms and therapeutic targets for AD. In this study, we summarized the recent progress about the functions of M1 and M2 microglia in AD pathogenesis and discussed underlying mechanisms.

## Characteristics of Microglia

### The Origin and Proliferation of Microglia

Microglial cells play vital roles in regulating the homeostasis of the central nervous system (CNS; Subhramanyam et al., 2019). However, the origin of microglia is still controversial. Earlier studies indicated that microglial cells descended from meningeal macrophages, which were invaded into the brain during the late stage of embryonic development (Alliot et al., 1999). Later, it was found that microglia in the parenchyma of the CNS displayed macrophage markers and originated from the hemopoietic system (Perry et al., 1985; Lawson et al., 1990; Gordon et al., 1992; Alliot et al., 1999). Alliot et al. (1999) reported that microglia were derived from its progenitors originating from the hemopoietic cells as early as embryonic day 8 (E8) in the yolk sac. The microglial progenitors migrated into the brain subsequently and increased the number fast (Alliot et al., 1999). Ginhoux et al. (2010) showed that adult microglial cells were originated from the original myeloid progenitors before E8. Kierdorf et al. (2013) also reported that microglia were originated from the c-Kit positive erythro myeloid progenitors as early as E8 in the yolk sac. Early studies have demonstrated that there were three waves of hematopoiesis in the yolk sac during the embryonic development of mice (Yoder, 2014). Except for the classical microglial progenitors mainly produced during the first wave of hematopoiesis in the yolk sac, there also existed a microglial subpopulation originated from the second wave of hematopoiesis in the yolk sac (De et al., 2018). Taken together, most of these studies indicated that microglia were originated from the yolk sac during development. However, further investigation is necessary to clarify which wave of hematopoiesis and which type of cells in the yolk sac were mainly responsible for the microglial origination.

The microglia isolated from adult postmortem cerebral cortex exhibited proliferative ability *in vitro* and maintained its phenotype (Guo et al., 2016). The colony-stimulating factor-1 receptor (CSF1R) was vital for the development of microglia (Ginhoux et al., 2010; Erblich et al., 2011). Microglia could be repopulated after the withdrawal of CSF1R antagonism PLX5622 in both adult and aged mice (O’Neil et al., 2018). Moreover, CSF1R inhibitor PLX3397 treatment resulted in a dramatic decrease in microglia number, while microglia were restored rapidly after the removal of PLX3397 in mice (Elmore et al., 2014). Elmore et al. (2014) revealed that the rapidly repopulated microglia were from the nestin-positive non-microglial progenitor cells, indicating that the newborn microglia were derived from the nestin-positive progenitors but not the remaining microglia. However, Huang et al. (2018) found that the repopulated microglial cells were not derived from the blood cells or astrocytes, neurons, and NG2 cells but from CX3CR1-positive microglia, demonstrating that the adult microglia could maintain its population through self-renewal. The inconformity of these two studies might result from the different protocols of drug treatment and different types of transgenic mice.

### Microglia Polarization

**FIGURE 1:**
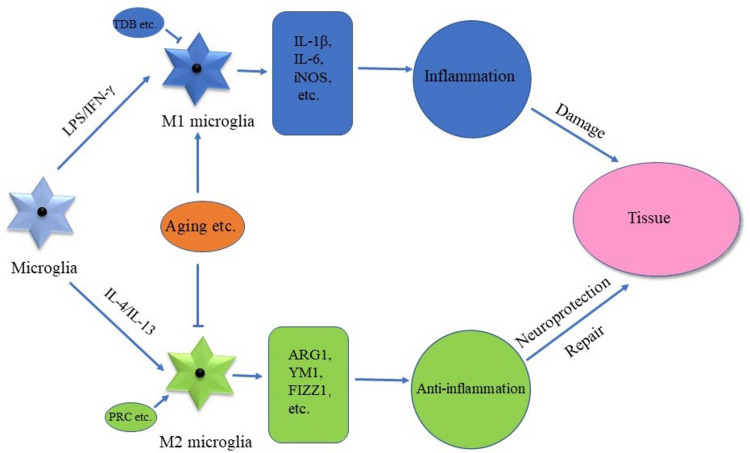
A paradigm of M1 and M2 phenotypes of microglial cells. Microglia activation is categorized into two phenotypes, namely, M1 phenotype and M2 phenotype. M1 microglia could be induced by lipopolysaccharide/interferon-γ (LPS/IFN-γ), leading to the increase of pro-inflammatory factors. M2 microglia could be induced by IL-4/IL-13, which leads to the increase of anti-inflammatory factors. Thus, M1 microglia contribute to the inflammatory response, while M2 microglia contribute to the neuroprotection and repair processes of tissues.

Growing evidence indicated that two types of activated microglia existed, namely, M1 phenotype and M2 phenotype (Wang Y. et al., 2019), although there are different opinions about the existence of different phenotypes of microglia activation (Ransohoff, 2016). M1 phenotype microglia were the classical form of microglial activation and contributed to the development of inflammatory responses especially in neurodegeneration (Tang and Le, 2016; Kwon and Koh, 2020). M1 microglia could be induced by lipopolysaccharide (LPS)/interferon-γ (IFN-γ; Tang and Le, 2016; Figure 1). M2 microglia were the alternative form of microglia activation and considered as the anti-inflammatory response phenotype, contributing to tissue repair and neuroprotection (Figure 1). Moreover, M2 microglia could be induced by interleukin-4 (IL-4)/IL-13 (Tang and Le, 2016; Kwon and Koh, 2020; Figure 1). In addition, the expression levels of pro-inflammatory markers were enhanced significantly in M1 microglia, while the expression levels of anti-inflammatory factors increased in M2 microglia (Lam et al., 2017; Kwon and Koh, 2020). As the pro-inflammatory factors were significantly upregulated in M1 microglia, the inflammation-associated factors such as IL-1β, IL-6, and inducible nitric oxide synthase (iNOS) were taken as the markers of M1 microglia (Jin et al., 2018; Figure 1). The expression of some relatively specific markers was upregulated in M2 microglia such as arginase 1 (ARG1), chitinase-3-like-3 (YM1, also called CHI3L3), and found in inflammatory zone 1 (FIZZ1; Jin et al., 2018; Veremeyko et al., 2018; Wu et al., 2019; Figure 1). Due to the diversity of diseases and different stages of diseases, it is essential to identify specific markers of microglial activation in various pathological conditions.

### Regulation of Microglia Polarization

Accumulated evidence showed that microglial polarization could be regulated. For example, PGC-1-related coactivator (PRC) was significantly increased in M2 microglia induced by IL-4 (Mou et al., 2015). Consistently, the overexpression of PRC increased the mRNA expression of M2 microglial markers such as ARG1, FIZZ1, and YM1, promoting microglia activation toward the M2 phenotype (Figure 1; Mou et al., 2015). Trehalose-6,6′-dibehenate (TDB) could decrease the expression of M1 microglial markers such as the proIL-1β and IL-6 induced by LPS (Mohanraj et al., 2019), while treatment with TDB also led to the significant increase of M2 microglia markers such as ARG1 and YM1/2 (Mohanraj et al., 2019; Figure 1). In addition, the dehydrocorydaline administration promoted the microglia toward the M2 phenotype in the spinal cord of a mouse model of bone cancer pain (Huo et al., 2018). Furthermore, TOPK, a mitogen-activated protein kinase, promoted the M2 microglial phenotype possibly through inhibiting the activity of histone deacetylases HDAC1 and HDAC2 (Han et al., 2018), while inhibiting H3K27me3 demethylase Jmjd3 enhanced the pro-inflammatory responses and suppressed the microglial M2 phenotype (Tang et al., 2014). It indicated that microglial polarization could be regulated by many signaling pathways.

Importantly, evidence indicated that aging plays a pivotal role in the balance of M1 and M2 phenotypes. Aging promoted the M1 phenotype with higher levels of pro-inflammatory factors such as IL-1β and tumor necrosis factor-α (TNF-α), while it decreased the activation of M2 microglia with a reduction of M2 markers such as ARG1, following peripheral surgery (Zhang Z. J. et al., 2019; Figure 1). When compared to 6-month-old mice, the expression of M1-related transcripts, such as S100A9 and CXCL13, tended to increase in 24-month-old mice treated with IL-1β, IL-12, and TNF-α, while an overall reduction of M2 microglia-associated markers such as ARG1, CHI3L3, and FIZZ1 was observed in 24-month-old mice treated with IL-4 and IL-13 cocktail. It indicated that aging facilitates the M1 phenotype but inhibits the M2 phenotype (Lee et al., 2013).

## Dysregulation of Microglia Polarization in Alzheimer’s Disease

**FIGURE 2:**
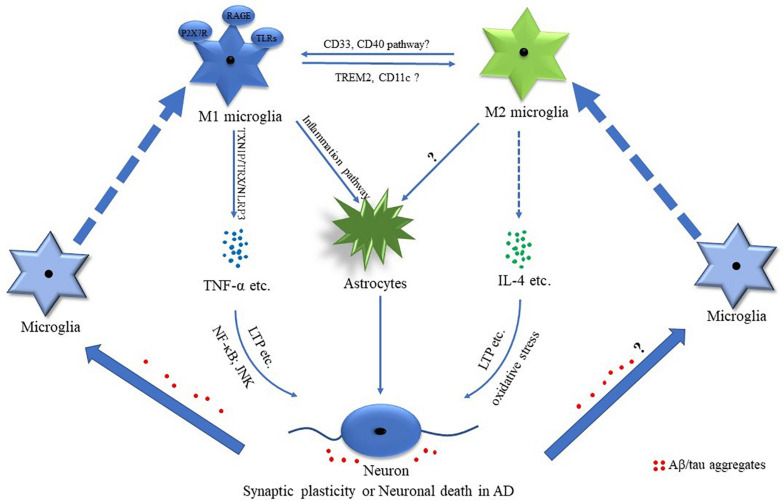
Potential mechanisms of M1 and M2 phenotypes of microglial cells in AD. Mounting evidence suggests that M1 and M2 microglia are involved in the pathological processes of Alzheimer’s disease (AD). The AD-associated pro-inflammatory and anti-inflammatory factors such as tumor necrosis factor-α (TNF-α) and interleukin-4 (IL-4) play important roles in regulating synaptic plasticity and neuronal death. Amyloid-β (Aβ)/tau aggregates are involved in regulating microglia-related inflammation.

Increased mRNA of TNF-α was observed in the frontal lobe cortex of patients with AD (Colton et al., 2006). The mRNA of CHI3L1 and CHI3L2, which are the two YM1 closely related genes, was robustly increased in the cortex of the patients with AD (Colton et al., 2006). It indicated that both classically activated microglial cells and alternatively activated microglia existed in AD brains (Figure 2). However, cell population-specific alteration of these markers was undefined. For example, the expression of CD40 was upregulated in microglia of AD brains (Togo et al., 2000), while CD40 ligand deficiency resulted in a significant reduction of TNF-α expression in cultured microglia of AD model mice (Tan et al., 1999). It indicated that the CD40-mediated pathway may play an important role in the M1 phenotype (Tan et al., 1999; Walker and Lue, 2015; Figure 2). Recent studies showed that activated microglia around amyloid plaques consisted of CD11c-positive and CD11c-negative subgroups in AD mice (Kamphuis et al., 2016). More interestingly, the transcriptional profiling analysis implied that CD11c-positive cells displayed increased immunosuppressive features counteracting the inflammatory response (Kamphuis et al., 2016). Hence, it was deduced that CD11c might be a marker of M2 microglia in AD (Figure 2).

Many susceptibility genes preferentially expressed in microglia of the aged human brain (e.g., *TREM2*, *CD33*, *SORL1*, and *INPP5D)* were associated with AD (Olah et al., 2018), while genetic analysis also indicated that various immune-related genes were associated with AD risk (Karch and Goate, 2015). It is highly supported that microglia may play a key role in AD pathogenesis (Karch and Goate, 2015; Katsumoto et al., 2018). For example, soluble triggering receptor expressed on myeloid cells 2 (sTREM2), a marker of microglia activation, was enhanced in cerebrospinal fluid at the stages of preclinical subjective cognitive decline, mild cognitive impairment (MCI), and AD dementia compared with healthy controls (Nordengen et al., 2019). In addition, TREM2 rare variants increased the risk of AD in Asian and European populations (Jonsson et al., 2013; Kleinberger et al., 2014; Jiang et al., 2016; Efthymiou and Goate, 2017). *TREM2* mRNA was enhanced in patients with AD. Increased TREM2 promoted ARG1 expression and reduced the nitric oxide (NO) production (Zhang et al., 2018; Figure 2). In addition, TREM2 promoted microglial phagocytosis (Kawabori et al., 2015; Zhang et al., 2018). Moreover, TREM2 deficiency did accelerate AD progress (Wang et al., 2015; Ma et al., 2016; Efthymiou and Goate, 2017), which might be associated with TREM2 deficiency-mediated downregulation of ARG1 in microglial cells (Zhang et al., 2018).

CD33 was mainly expressed in microglia and macrophages in the brain (Zhao, 2019). It was reduced in peripheral mononuclear cells of patients with AD, while it was increased in the frontal cortex of patients with AD (Griciuc et al., 2013; Hu et al., 2014). Consistently, the number of CD33-positive microglia markedly increased in the cortex of AD cases (Griciuc et al., 2013). CD33 knockout led to the increase of inflammasome genes and anti-inflammatory gene expression in AD mice (Griciuc et al., 2019), while a recent study further demonstrated that CD33 knockdown significantly decreased the pro-inflammatory-related transcripts in AD mice (Griciuc et al., 2020; Figure 2).

P2X7 receptor (P2X7R), which is a purinergic receptor, was significantly increased in microglial cells of the patients with AD, which was associated with the pathology of AD. The expression of P2X7R was higher in microglia of AD mice at both the advanced and the late stages, while no significant difference was detected at the early stage (Lee et al., 2011; Martinez-Frailes et al., 2019). The activation of P2X7R promoted microglia migration toward the senile plaques, while the inhibition of P2X7R promoted the phagocytosis of microglia (Martinez-Frailes et al., 2019). Moreover, P2X7R was involved in the degenerative neuron-induced microglial activation contributing to the activation of astrocytes resulting in the amplification of the neuroinflammation and neuronal damage (Yiangou et al., 2006; Glass et al., 2010). Consistently, P2X7R inhibition significantly reduced the expression of IL-1β in spinal cord microglia of a rat pain model (Zhou et al., 2019). It indicated that P2X7R may be a key player of M1 microglia (Figure 2).

Notably, microglia not only contributed to neuronal death by releasing pro-inflammatory cytokines but also exerted neuroprotective function *via* phagocytosis in AD (Pourbadie et al., 2018; Rangaraju et al., 2018). Specifically, the proportions of CD44^–^CXCR4^+^ and CD44^+^CXCR4^–^ microglia were altered with age in AD mice. It suggested that aging may affect the profiles of M1 and M2 microglia in AD as CD44 and CXCR4 contributed to the pro-inflammatory and anti-inflammatory processes, respectively (Rangaraju et al., 2018). Moreover, it was indicated that M2 microglia switched into the M1 phenotype at the advanced stage of AD (Jimenez et al., 2008). However, the regulation of M1/M2 microglia in AD remains elusive.

## The Role of Microglia Polarization in the Pathogenesis of Alzheimer’s Disease

### Mutual Regulation Between Aβ Aggregates and Microglial Polarization

Amyloid-β was associated with the microglia activation in AD (Glass et al., 2010). The pathological analysis showed that activated microglia were accumulated around the plaques (Yin et al., 2017). Aggregated Aβ-induced M1 microglia were partially mediated by the receptor for advanced glycoxidation end-products (RAGE) and toll-like receptors (TLRs) (Figure 2), while the pro-inflammatory cytokines of M1 microglia further led to the activation of astrocytes, contributing to neuronal loss in AD (Glass et al., 2010; Desale and Chinnathambi, 2020; Figure 2). Early studies demonstrated that concentrated fibrillar Aβ induced the microglia/monocyte activation with the increased expression of iNOS, which was TNF-α dependent, contributing to neuronal death (Combs et al., 2001; Chen et al., 2005). Recent studies showed that Aβ-induced pro-inflammatory factors such as IL-6 and TNF-α were possibly medicated by thioredoxin-interacting protein (TXNIP)/thioredoxin (TRX)/NOD-like receptor pyrin domain-containing protein 3 (NLRP3) pathway in microglial cells (Feng and Zhang, 2019; Figure 2). Moreover, Aβ could induce the activation of NLRP3 inflammasome in microglia, contributing to the activation of microglia toward M1 polarization (Halle et al., 2008; Liang et al., 2020; Zhang et al., 2020). Another study showed that Aβ led to the enhancement of pro-inflammatory cytokines such as IL-1β and IL-6 *via* regulating the homeostasis of ornithine decarboxylase and antizyme (Cheng et al., 2019). In addition, Cui et al. (2019) reported that enhanced NF-E2-related factor 2 (Nrf2)/Kelch-like ECH-associated protein 1 (Keap1) signaling significantly attenuated the pro-inflammatory responses and oxidative stress induced by Aβ. These results indicated that Aβ could directly induce or potentiate the M1 activation of microglia.

As the immune cells maintain the tissue homeostasis in the CNS, microglia cleared the debris and misfolded proteins by phagocytosis in the brain (Kettenmann et al., 2011; Zhou et al., 2020). It was reported that microglia cleared the Aβ *via* phagocytosis and proteases-mediated degradation (Lopez-Valdes and Martinez-Coria, 2016). Chronically, the activation of M1 microglia released the cytotoxic factors and might have deficits in the clearance of Aβ aggregates, accelerating the progress of AD (Glass et al., 2010; Lian et al., 2016; Zhang X. et al., 2019). The inactivation of CD33 promoted microglial uptake of Aβ, while the enhancement of CD33 significantly inhibited the uptake of Aβ by microglia (Griciuc et al., 2013). CD33 promoted the development of Aβ aggregates, contributing to AD pathology (Griciuc et al., 2013). In addition, TLR2 deficiency led to the enhancement of Aβ phagocytosis (Liu et al., 2012), while TLR2 knockout promoted M1 microglia to M2 microglia in bone marrow chimeric APP transgenic mice (Liu et al., 2012). Deferoxamine promoted the M2 activation of microglia and significantly reduced the Aβ deposits in AD mice (Zhang and He, 2017). Sarsasapogenin-AA13 significantly alleviated Aβ-induced cognitive deficiency possibly through reducing the M1 activation, increasing the M2 activation, and enhancing Aβ clearance in mice (Huang et al., 2017). It was suggested that treadmill exercise enhanced the M2 activation and decreased the M1 activation of microglia, contributing to the decrease of Aβ deposition and improvement of cognition in AD mice (Zhang X. et al., 2019). These results indicated that microglial polarization has a significant effect on Aβ deposition, i.e., M2 microglia might have a strong ability to clear Aβ. However, further investigation is needed to elucidate the association between microglial activation and Aβ clearance.

### Mutual Regulation Between Tau Aggregates and Microglial Polarization

Increasing evidence indicated that tau was closely associated with microglia activation. The tau-induced activation of microglia was medicated by the NLRP3 inflammasome, contributing to the release of IL-1β (Ising et al., 2019). NLRP3 knockout greatly inhibited tau hyperphosphorylation induced by brain homogenates of APP/PS1 mice, indicating the vital roles of NLRP3 in tau pathology (Ising et al., 2019). Ionized calcium-binding adaptor molecule-1 (Iba1) was often taken as the marker of microglia activation following inflammatory stimulation (Hoogland et al., 2015). Tau aggregates could spread the localization of Iba1 to the cell membrane, and tau oligomer also induced the increase of cytosolic Iba1 levels in microglia, indicating that tau could drive the pro-inflammatory activation of microglia in AD (Das et al., 2020; Figure 2). On the other hand, the pro-inflammatory cytokines released from the activated microglia also contributed to the regulation of tau phosphorylation and the formation of tau aggregates (Li et al., 2003; Barron et al., 2017; Leyns and Holtzman, 2017). For example, IL-1β secreted by microglia triggered tau phosphorylation through the p38 mitogen-activated protein kinase (MAPK) pathway (Li et al., 2003; Barron et al., 2017; Desale and Chinnathambi, 2020). TNF-α induced the hyperphosphorylation of tau while the long-term treatment of TNF-α led to a marked decrease of tau hyperphosphorylation in AD mice (Janelsins et al., 2008). However, the immunohistochemical analysis showed that another pro-inflammatory factor IFN-γ significantly decreased the levels of hyperphosphorylated tau in the brain of AD mice, indicating the potential roles of IFN-γ in tau hyperphosphorylation (Mastrangelo et al., 2009). Moreover, it was suggested that microglia exerted the cleaning function to clear tau oligomers by phagocytosis in AD (Das et al., 2020). It indicated that tau aggregates and M1 microglial activation are possibly mutually regulated.

### Synaptic Dysfunction and Neuronal Death Mediated by Microglia Polarization

Emerging evidence demonstrated that the AD-associated pro-inflammatory factors such as IL-1β and TNF-α played important roles in synaptic dysfunction and neuronal death (Gaur and Agnihotri, 2015; Pettigrew et al., 2016; Rincon-Lopez et al., 2017; Rizzo et al., 2018; Xiao et al., 2020). For example, increased IL-1β was observed during the long-term potentiation (LTP), while IL-1 receptor blockade led to the impairment of LTP in the hippocampus, indicating that IL-1β is implicated in the regulation of synaptic plasticity (Schneider et al., 1998; Coogan et al., 1999; Avital et al., 2003). Moreover, IL-1β promoted neuronal death in the hippocampus of developing rats with status epilepticus (Rincon-Lopez et al., 2017). In addition, TNF-α mediated polyinosinic-polycytidylic acid [Poly(I:C)]-induced elimination and formation of the dendritic spine in wild-type mice (Garre et al., 2017). Consistently, dominant-negative TNF-α significantly inhibited dendritic spine elimination and formation in the somatosensory cortex of experimental autoimmune encephalomyelitis (EAE) mice (Yang et al., 2013). TNF-α significantly increased the LTP levels of the hippocampus (Pettigrew et al., 2016; Figure 2). Furthermore, TNF-α induced neuronal apoptosis possibly through the upregulation of c-Jun N-terminal kinase (JNK) activation and increased nuclear factor kappa B (NF-κB) p65 and iNOS (Yang et al., 2002; Kraft et al., 2009; Xiao et al., 2020). The aforementioned evidence indicated the important role of TNF-α in the regulation of synaptic plasticity and neuronal death (Figure 2).

Anti-inflammatory cytokines were implicated in synaptic plasticity and neuronal death. A significant increase of IL-4 was observed in patients with AD (King et al., 2018), which played important roles in the protection of synaptic plasticity and neuronal death (Maher et al., 2005; Bhattarai et al., 2016; Hernandez-Espinosa et al., 2019; Jeong et al., 2019; Taipa et al., 2019). Maher et al. (2005) reported that the decrease of IL-4-related signaling contributed to the LTP deficits in the aged rat (Figure 2), while IL-4 and IL-10 administration significantly decreased the neuronal injury induced by excitotoxic damage in wild-type animals (Hernandez-Espinosa et al., 2019). However, IL-4 aggravated the neuronal death induced by prothrombin kringle-2 (pKr-2) possibly through regulating the oxidative stress (Jeong et al., 2019; Figure 2). All these results implicated that the AD-related pro-inflammatory and anti-inflammatory cytokines were involved in modulating synaptic plasticity and neuronal death.

Taken together, besides involving in the regulation of the synaptic dysfunction and neuronal death by the inflammatory-related factors, the activated microglia could also affect the pathological process of AD through regulating Aβ and tau deposition. It is noteworthy that different pro-inflammatory cytokines or anti-inflammatory factors might have their unique function in Aβ and tau pathology in AD.

## The Therapeutic Potential of Targeting Microglia Polarization

The Aβ and tau aggregates mainly induced M1 microglia (Glass et al., 2010; Das et al., 2020), while the inflammation mediated by microglia was involved in the formation of Aβ and tau aggregates, as well as the synaptic and neuronal loss contributing to the neurodegeneration (Jimenez et al., 2008; Glass et al., 2010; Gaur and Agnihotri, 2015; Lian et al., 2016; Pettigrew et al., 2016; Rincon-Lopez et al., 2017; Rizzo et al., 2018; Zhang X. et al., 2019; Xiao et al., 2020). Thus, it was crucial to clarify the molecular mechanisms of microglial activation in AD, which will help to find the efficient drugs that targeted the microglial activation such as the inhibition of pro-inflammation or the promotion of anti-inflammation. For example, CD33 inactivation promoted the uptake of Aβ by microglia (Griciuc et al., 2013). Consistently, CD33 knockdown significantly decreased the pro-inflammatory-related transcripts and Aβ plaque in AD mice at an early age (Griciuc et al., 2020). Thus, CD33 might be a potential target for AD treatment. In addition, P2X7R was indicated as a key player in M1 microglia (Zhou et al., 2019). Targeting P2X7R might be beneficial for AD treatment by inhibiting M1 microglia. Moreover, increased TREM2 promoted M2 phenotype, indicating that increasing the TREM2 activity may be a potential approach for AD treatment by promoting M2 microglia (Zhang et al., 2018). Furthermore, TLR2 knockout contributed to the transition from M1 to M2 microglia (Liu et al., 2012). It suggested that targeting TLR2 might be a potential approach for AD treatment by promoting M1 to M2 shift. Therefore, efficiently targeting the abovementioned or more candidates facilitating the shift from M1 to M2 may have therapeutic potential for AD.

## Conclusion

Mounting evidence has shown that the microglia-associated neuroinflammation was one of the major hallmarks of AD, while the microglial activation was correlated with the progress of AD. M1 and M2 microglia, i.e., pro-inflammation and anti-inflammation phenotypes, played differential roles in AD pathogenesis although precise characteristics and regulation of them still need to be fully elucidated. Balancing M1 and M2 microglia or promoting the shift from M1 to M2 might have therapeutic potential for AD treatment.

## Author Contributions

QW and HY wrote the manuscript. WL, BY, HC, and ZX contributed to the revision of the manuscript. YW conducted the editing of the manuscript. All authors approved the final manuscript.

## Conflict of Interest

The authors declare that the research was conducted in the absence of any commercial or financial relationships that could be construed as a potential conflict of interest.

## Publisher’s Note

All claims expressed in this article are solely those of the authors and do not necessarily represent those of their affiliated organizations, or those of the publisher, the editors and the reviewers. Any product that may be evaluated in this article, or claim that may be made by its manufacturer, is not guaranteed or endorsed by the publisher.
